# Association between body roundness index and urinary incontinence in American adults: A cross-sectional study of NHANES 2001 to 2018

**DOI:** 10.1097/MD.0000000000047582

**Published:** 2026-02-06

**Authors:** Tianyu Huang, Qiulu Huang, Haote Chen, Liyang Chang, Yanjuan Li, Mei Yang

**Affiliations:** a Hangzhou TCM Hospital of Zhejiang Chinese Medical University (Hangzhou Hospital of Traditional Chinese Medicine), Hangzhou, Zhejiang, China; b Department of Nursing, Hangzhou TCM Hospital Affiliated to Zhejiang Chinese Medicine University, Hangzhou, Zhejiang, China; c Department of Urology, Hangzhou TCM Hospital Affiliated to Zhejiang Chinese Medicine University, Hangzhou, Zhejiang, China; d Hangzhou Municipal Health Commission, Hangzhou, Zhejiang, China.

**Keywords:** body roundness index, mixed urinary incontinence, NHANES, stress urinary incontinence, urge urinary incontinence, urinary incontinence

## Abstract

This study aims to investigate the association between Body Roundness Index (BRI) and urinary incontinence (UI) subtypes (stress [SUI], urge [UUI], and mixed [MUI]). This cross-sectional study analyzed NHANES 2001 to 2018 data from 28,639 participants aged ≥20 years. Participants were stratified by BRI quartiles. Multivariable logistic regression examined associations between BRI and UI subtypes. Restricted cubic spline analysis explored nonlinear relationships. Threshold effect analysis identified inflection points. In fully adjusted models, each unit increase in BRI increased risk by 12% for SUI (OR = 1.12, 95% CI: 1.10–1.14) and UUI (OR = 1.12, 95% CI: 1.10–1.13), and 13% for MUI (OR = 1.13, 95% CI: 1.11–1.16) (all *P* <.0001). Compared to the lowest quartile, the highest quartile showed elevated risks: 109% for SUI (OR = 2.09, 95% CI: 1.88–2.32), 81% for UUI (OR = 1.81, 95% CI: 1.64–2.01), and 113% for MUI (OR = 2.13, 95% CI: 1.85–2.47). Threshold analysis revealed inflection points at BRI = 2.72 for UUI and BRI = 7.8 for SUI and MUI. Below the UUI threshold, association was negative (OR = 0.71, 95% CI: 0.55–0.91); above it, positive (OR = 1.13, 95% CI: 1.11–1.15). Poverty-income ratio moderated the BRI-SUI relationship (*P* = .0218). BRI demonstrated significant dose-response associations with all UI subtypes in fully adjusted models. Quartile analysis revealed that the highest BRI quartile showed significant associations across subtypes, with MUI displaying the strongest association. Nonlinear threshold analyses identified subtype-specific inflection points, indicating distinct association patterns for different UI subtypes.

## 1. Introduction

Urinary incontinence (UI) is a common urological disorder characterized by involuntary urine leakage, encompassing 3 types: UUI, SUI, and MUI.^[1]^ Relevant studies indicate that the prevalence of UI in males ranges from approximately 1% to 40%, while in females it is about twice that of males, ranging from 25% to 45%. Furthermore, the incidence of UI increases significantly with age.^[2]^ This condition not only severely impacts patients’ quality of life but also contributes to mental health issues and increased healthcare economic burdens.^[2,3]^ Therefore, identifying risk factors for UI and implementing early interventions are imperative.

Obesity has now evolved into a global health concern and serves as a risk factor for numerous chronic diseases.^[4]^ An increasing amount of research indicates that obesity is closely associated with the occurrence and exacerbation of UI.^[5,6]^ The underlying mechanisms may involve increased intra-abdominal pressure due to visceral fat accumulation, as well as impaired pelvic floor muscle function and pelvic nerve innervation.^[7,8]^ Compared to general obesity, abdominal obesity may exhibit a stronger correlation with UI development.^[9]^ However, traditional obesity assessment metrics such as body mass index (BMI) and waist circumference (WC) may fail to adequately reflect body fat distribution. Body roundness index (BRI), developed by Thomas et al, estimates body fat percentage by integrating height and WC data. This index demonstrates enhanced accuracy in predicting visceral adipose tissue and total body fat percentage, offering particular advantages in assessing abdominal obesity.^[10,11]^ Emerging evidence reveals that elevated BRI levels significantly correlate with increased risks of several urological disorders, including benign prostatic hyperplasia,^[12]^ nephrolithiasis,^[13]^ and overactive bladder.^[14]^ Nevertheless, no studies to date have investigated the relationship between UI and BRI.

Therefore, this study conducts a cross-sectional analysis using data from the NHANES to explore the association between BRI and UI, aiming to provide a new perspective for early identification and targeted intervention in high-risk populations.

## 2. Materials and methods

### 2.1. Study design and population

**Figure 1.**
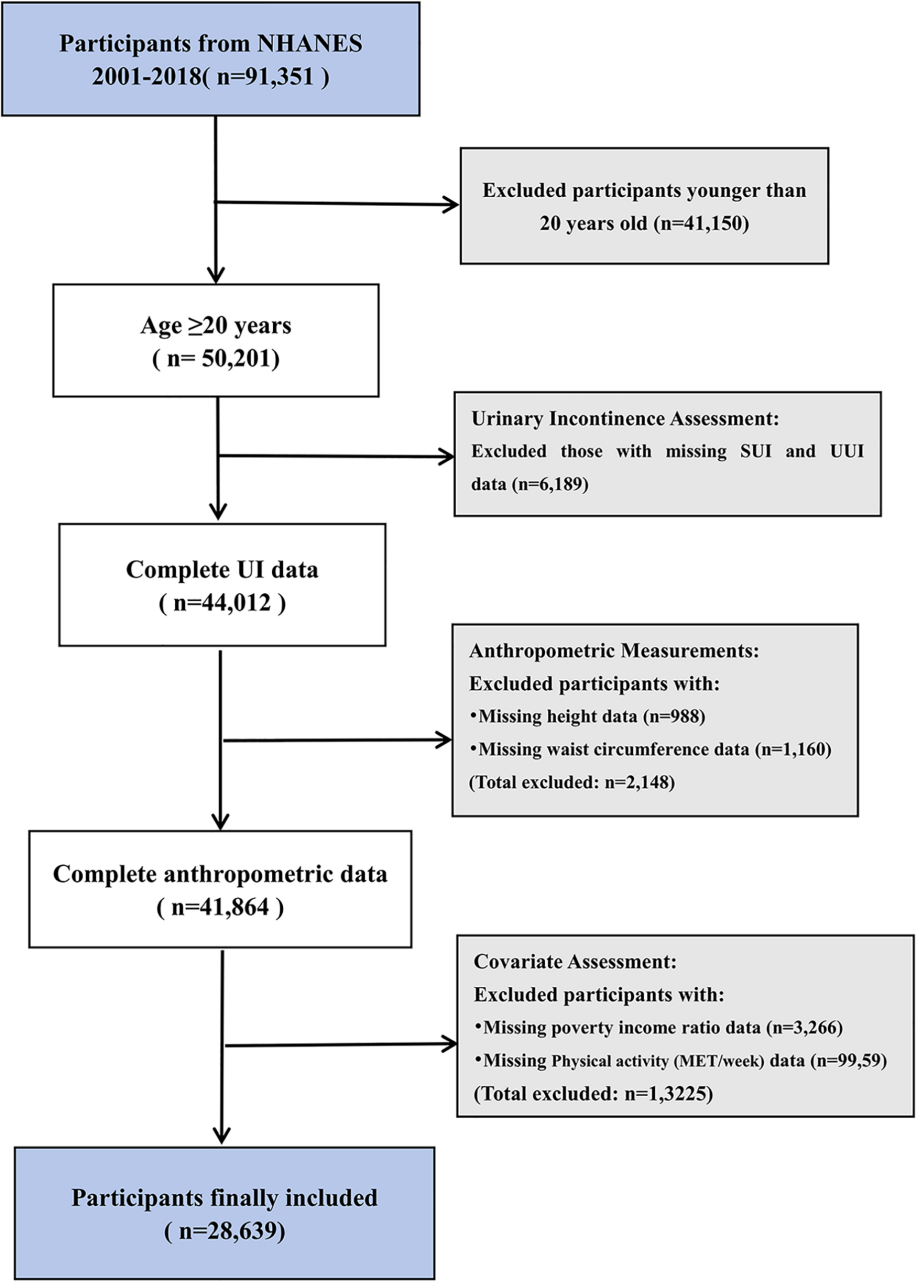
Study flow chart of participant involvement. MET = metabolic equivalent of task, NHANES = National Health and Nutrition Examination Survey, SUI = stress urinary incontinence, UI = urinary incontinence, UUI = urge urinary incontinence.

This study used data from the NHANES. NHANES is a comprehensive nationwide survey conducted by the National Center for Health Statistics to assess the health and nutritional status of the US population.^[15]^ All participants signed a written informed consent form, and the NHANES survey was approved by the Research Ethics Review Board of the National Center for Health Statistics. To ensure transparency and consistency in study design and reporting, this study adhered to the cross-sectional study guidelines established by the Strengthening the Reporting of Observational Studies in Epidemiology (STROBE).^[16]^ We analyzed NHANES data spanning 9 survey cycles (2001–2018) because these 9 2-year cycles contained questionnaire data on UI, which initially included 91,351 participants. We sequentially excluded participants based on the following criteria: [1] age < 20 years (n = 41,150); [2] missing SUI and UUI data (n = 6189); [3] missing anthropometric data for BRI calculation, including height (n = 988) and WC (n = 1160); and [4] missing covariate data, including poverty-income ratio (PIR) (n = 3266) and Physical activity (MET/week) (n = 9959) (Fig. 1). After applying these exclusion criteria, the final analytical sample comprised 28,639 participants aged ≥ 20 years with complete data on UI, anthropometric measurements, and all relevant covariates.

### 2.2. Assessment of BRI

The exposure variable of this study was BRI, calculated from WC and height,^[10]^ and measured by trained personnel at mobile examination centers. Due to the lack of established cutoff values, BRI was categorized into quartiles to investigate its association with UI. The formula is as follows:


$$BRI=364.2-365.5\times \sqrt{1-{\left(\frac{WC(m)÷2\pi }{0.5\times Height(m)}\right)}^{2}}$$

### 2.3. Assessment of UI

The outcome variable of this study is UI, including SUI, UUI, and MUI, assessed through 2 specific questions in the “Kidney Conditions – Urology” questionnaire of the NHANES database.^[17]^ SUI was considered present if the participant answered “yes” to the question: “During the past 12 months, have you leaked or lost control of even a small amount of urine with an activity like coughing, lifting, or exercise?” UUI was considered present if the participant answered “yes” to the question: “During the past 12 months, have you leaked or lost control of even a small amount of urine with an urge or pressure to urinate, and you could not get to the toilet fast enough?” The co-occurrence of both SUI and UUI was classified as MUI.^[18]^

### 2.4. Covariates

Drawing on prior research, this study extracted from the NHANES database as many covariates as possible that may influence BRI or UI. First, demographic characteristics comprised age (20–44, 45–64, ≥65 years), sex, race (non-Hispanic White, non-Hispanic Black, Mexican American, other Hispanic, other races), marital status (married/living with partner, widowed/divorced/separated, never married), education level (below high school, high school, above high school), and PIR (<1.3, 1.3–3.4, ≥3.5). In addition, several lifestyle factors were included as covariates: smoking status (never, former, now), alcohol use (defined as the average number of alcoholic drinks per day over the past 12 months), and physical activity (categorized into low, medium, and high tertiles based on weekly total metabolic equivalents). Previous studies have demonstrated significant associations between these lifestyle factors and urinary symptoms.^[19–21]^ Finally, 3 chronic conditions (hypertension, diabetes, and hyperlipidemia) related to UI were also included as covariates.^[22,23]^

### 2.5. Statistical analysis

Baseline characteristics were presented across BRI quartiles, with categorical variables expressed as frequencies and percentages, and continuous variables as means ± standard deviations or medians with interquartile ranges depending on their distribution. Differences across quartiles were evaluated using chi-square tests or Fisher exact tests (when expected frequency < 5) for categorical variables and Kruskal-Wallis tests for continuous variables. To evaluate the association between BRI and UI subtypes, we constructed 3 progressively adjusted logistic regression models: Model 1 (unadjusted), Model 2 (adjusted for age, gender, and race), and Model 3 (fully adjusted for all covariates, including age, gender, race, marital status, education level, PIR, smoking status, alcohol use, physical activity, hypertension, diabetes, and hyperlipidemia). Results were expressed as odds ratios with 95% confidence intervals. For quartile analysis, P for trend was calculated by treating BRI quartiles as a continuous variable. To explore potential nonlinear relationships, we conducted restricted cubic spline analyses with 3 knots, followed by threshold effect analysis using 2-piecewise linear regression models. The optimal inflection point was determined using a log-likelihood ratio test comparing the 1-line linear model with the 2-piecewise model. We performed stratified analyses to assess the consistency of associations across subgroups defined by gender, age, PIR, smoking status, alcohol consumption, physical activity, hypertension, diabetes, and hyperlipidemia. Interaction terms were tested in the fully adjusted models to determine whether the association between BRI and UI subtypes differed significantly across these subgroups. All statistical analyses were performed using EmpowerStats (Version 2.0) and Stata for supplementary analyses. Two-sided P-values < 0.05 were considered statistically significant.

## 3. Result

### 3.1. Baseline characteristics

A total of 28,639 participants were stratified by BRI quartiles (Q1: <3.68, Q2: 3.68–4.92, Q3: 4.92–6.41, Q4: >6.41) (Table 1). All baseline characteristics differed significantly across quartiles (*P* < .001). Higher BRI quartiles were associated with older age (≥45 years: 32.75% in Q1 vs 61.73% in Q4), female predominance (45.89% in Q1 vs 59.39% in Q4), lower educational attainment (above high school: 64.19% in Q1 vs 50.11% in Q4), and lower income levels (PIR ≥ 3.5: 38.09% in Q1 vs 27.65% in Q4). Participants with higher BRI showed lower rates of current smoking and heavy alcohol consumption, along with decreased physical activity levels. Most notably, the prevalence of cardiometabolic conditions increased substantially across BRI quartiles, including hypertension (18.48% in Q1 vs 55.67% in Q4), diabetes (3.52% in Q1 vs 29.47% in Q4), and hyperlipidemia (44.62% in Q1 vs 80.56% in Q4). Similarly, UI prevalence increased with higher BRI (SUI: 14.37% vs 32.00%; UUI: 12.21% vs 29.39%; MUI: 4.86% vs 15.28% for Q1 vs Q4, respectively).

**Table 1:** Baseline characteristics of the study population according to quartiles of BRI.

BRI	Q1 (<3.68)	Q2 (≥3.68–4.922)	Q3 (≥4.922–6.41)	Q4 (≥6.41)	*P*-value
	n = 7160	n = 7159	n = 7160	n = 7160	
Age (year), n(%)					
20–44	4815 (67.25)	3310 (46.24)	2678 (37.40)	2740 (38.27)	<.001
45–64	1669 (23.31)	2454 (34.28)	2638 (36.84)	2688 (37.54)	
≥65	676 (9.44)	1395 (19.49)	1844 (25.75)	1732 (24.19)	
Gender, n(%)					
Male	3874 (54.11)	4172 (58.28)	3984 (55.64)	2908 (40.61)	<.001
Female	3286 (45.89)	2987 (41.72)	3176 (44.36)	4252 (59.39)	
Race, n(%)					
Non-Hispanic White	3658 (51.09)	3485 (48.68)	3430 (47.91)	3242 (45.28)	<.001
Non-Hispanic Black	1533 (21.41)	1224 (17.10)	1250 (17.46)	1634 (22.82)	
Mexican American	577 (8.06)	1065 (14.88)	1327 (18.53)	1337 (18.67)	
Other Hispanic	414 (5.78)	561 (7.84)	600 (8.38)	583 (8.14)	
Other Races	978 (13.66)	824 (11.51)	553 (7.72)	364 (5.08)	
Marital status, n(%)					
Married or Living with Partner	3859 (53.93)	4754 (66.42)	4779 (66.76)	4264 (59.56)	<.001
Widowed, divorced, or separated	1045 (14.60)	1278 (17.86)	1479 (20.66)	1760 (24.58)	
Never married	2252 (31.47)	1125 (15.72)	900 (12.57)	1135 (15.85)	
Education level, n(%)					
Below high school	291 (4.07)	565 (7.90)	732 (10.23)	714 (9.97)	<.001
High school	2272 (31.75)	2366 (33.07)	2599 (36.32)	2857 (39.91)	
Above high school	4594 (64.19)	4223 (59.03)	3825 (53.45)	3587 (50.11)	
Poverty-income ratio, n(%)					
<1.3	1937 (27.05)	1754 (24.50)	1858 (25.95)	2369 (33.09)	<.001
1.3–3.4	2496 (34.86)	2610 (36.46)	2814 (39.30)	2811 (39.26)	
≥3.5	2727 (38.09)	2795 (39.04)	2488 (34.75)	1980 (27.65)	
Smoking status, n(%)					
Never	4011 (56.04)	3905 (54.58)	3688 (51.52)	3862 (53.97)	<.001
Forme	1189 (16.61)	1747 (24.42)	2139 (29.88)	2024 (28.28)	
Now	1957 (27.34)	1503 (21.01)	1331 (18.59)	1270 (17.75)	
Alcohol use, n(%)					
Never	748 (10.60)	767 (10.92)	874 (12.49)	1017 (14.67)	<.001
Former	686 (9.72)	953 (13.57)	1180 (16.86)	1394 (20.11)	
Mild	2554 (36.19)	2667 (37.96)	2475 (35.37)	2152 (31.04)	
Moderate	1334 (18.90)	1135 (16.16)	1070 (15.29)	1049 (15.13)	
Heavy	1736 (24.60)	1503 (21.40)	1398 (19.98)	1320 (19.04)	
Physical activity (MET/wk), n(%)					
Low	2167 (30.27)	2341 (32.70)	2439 (34.06)	2557 (35.71)	<.001
Middle	2343 (32.72)	2421 (33.82)	2434 (33.99)	2385 (33.31)	
High	2650 (37.01)	2397 (33.48)	2287 (31.94)	2218 (30.98)	
Hypertension, n(%)					
No	5836 (81.52)	4740 (66.23)	3912 (54.65)	3174 (44.33)	<.001
Yes	1323 (18.48)	2417 (33.77)	3246 (45.35)	3986 (55.67)	
Diabetes, n(%)					
No	6558 (92.77)	5835 (83.23)	5179 (74.42)	4219 (61.25)	<.001
Yes	249 (3.52)	693 (9.88)	1168 (16.78)	2030 (29.47)	
Dysglycemia	262 (3.71)	483 (6.89)	612 (8.79)	639 (9.28)	
Hyperlipidemia, n(%)					
No	3965 (55.38)	2171 (30.33)	1538 (21.48)	1392 (19.44)	<.001
Yes	3195 (44.62)	4988 (69.67)	5622 (78.52)	5767 (80.56)	
SUI, n(%)					
No	6131 (85.63)	5882 (82.16)	5561 (77.67)	4869 (68.00)	<.001
Yes	1029 (14.37)	1277 (17.84)	1599 (22.33)	2291 (32.00)	
UUI, n(%)					
No	6286 (87.79)	5971 (83.41)	5691 (79.48)	5056 (70.61)	<.001
Yes	874 (12.21)	1188 (16.59)	1469 (20.52)	2104 (29.39)	
MUI, n(%)					
No	6812 (95.14)	6697 (93.55)	6509 (90.91)	6066 (84.72)	<.001
Yes	348 (4.86)	462 (6.45)	651 (9.09)	1094 (15.28)	

BRI quartile ranges: Q1: <3.68, Q2: 3.68–4.92, Q3: 4.92–6.41, Q4: >6.41. *P*-values for all categorical variables were < 0.001 by chi-square test or Fisher exact test (when theoretical frequency < 10); continuous variables were tested by Kruskal-Wallis test. Data are presented as n(%) or mean ± standard deviation.

BRI = body roundness index, MET = metabolic equivalent of task, MUI = mixed urinary incontinence, SUI = stress urinary incontinence, UUI = urge urinary incontinence.

### 3.2. The association between BRI and the risk of UI

To explore the relationship between BRI and UI, we employed 3 models for logistic regression analysis (Table 2). BRI demonstrated significant dose-dependent associations with all UI subtypes across progressively adjusted models. In the fully adjusted model (Model 3), each unit increase in BRI was associated with 12% higher odds of both SUI (OR = 1.12, 95% CI: 1.10–1.14) and UUI (OR = 1.12, 95% CI: 1.10–1.13), and 13% higher odds of MUI (OR = 1.13, 95% CI: 1.11–1.16) (all *P* < .0001). Quartile analysis revealed pronounced dose-response relationships for all subtypes (P for trend < 0.0001). Compared to the lowest BRI quartile (Q1), participants in the highest quartile (Q4) showed substantially elevated odds of SUI (OR = 2.09, 95% CI: 1.88–2.32), UUI (OR = 1.81, 95% CI: 1.64–2.01), and MUI (OR = 2.13, 95% CI: 1.85–2.47), representing approximately 109%, 81%, and 113% increased risk, respectively. Notably, MUI demonstrated the strongest association with BRI, with significant risk elevation beginning at Q3 (OR = 1.46, 95% CI: 1.26–1.70), while SUI showed consistent risk elevation from Q2 onwards (OR = 1.28, 95% CI: 1.15–1.42). For UUI, significant associations emerged from Q2 (OR = 1.12, 95% CI: 1.01–1.24, *P* = .0298) with progressive risk increases at higher quartiles. The magnitude of these associations remained robust after comprehensive adjustment for potential confounders.

**Table 2:** Multivariable logistic regression analysis of the association between BRI and UI subtypes.

Characteristics	Model 1		Model 2		Model 3	
	OR (95% CI)	*P*-value	OR (95% CI)	*P*-value	OR (95% CI)	*P*-value
SUI						
Continuous	1.18 (1.17–1.19)	<.0001	1.12 (1.11–1.14)	<.0001	1.12 (1.10–1.14)	<.0001
Categories						
Q1	Reference	–	Reference	–	Reference	–
Q2	1.29 (1.18–1.41)	<.0001	1.30 (1.18–1.44)	<.0001	1.28 (1.15–1.42)	<.0001
Q3	1.71 (1.57–1.87)	<.0001	1.64 (1.48–1.81)	<.0001	1.59 (1.43–1.76)	<.0001
Q4	2.80 (2.58–3.04)	<.0001	2.15 (1.95–2.36)	<.0001	2.09 (1.88–2.32)	<.0001
*P* for trend	<.0001	–	<.0001	–	<.0001	–
UUI						
Continuous	1.18 (1.17–1.20)	<.0001	1.12 (1.10–1.13)	<.0001	1.12 (1.10–1.13)	<.0001
Categories						
Q1	Reference	–	Reference	–	Reference	–
Q2	1.43 (1.30–1.57)	<.0001	1.09 (0.99–1.21)	.0817	1.12 (1.01–1.24)	.0298
Q3	1.86 (1.69–2.03)	<.0001	1.23 (1.11–1.36)	<.0001	1.26 (1.13–1.39)	<.0001
Q4	2.99 (2.74–3.27)	<.0001	1.82 (1.66–2.00)	<.0001	1.81 (1.64–2.01)	<.0001
*P* for trend	<.0001	–	<.0001	–	<.0001	–
MUI						
Continuous	1.21 (1.19, 1.23)	<.0001	1.14 (1.12, 1.16)	<.0001	1.13 (1.11, 1.16)	<.0001
Categories						
Q1	Reference		Reference		Reference	
Q2	1.35 (1.17–1.56)	<.0001	1.15 (0.99–1.34)	.0639	1.14 (0.98–1.33)	.0912
Q3	1.96 (1.71–2.24)	<.0001	1.49 (1.29–1.72)	<.0001	1.46 (1.26–1.70)	<.0001
Q4	3.53 (3.11–4.00)	<.0001	2.24 (1.96–2.57)	<.0001	2.13 (1.85–2.47)	<.0001
*P* for trend	<.0001	–	<.0001	–	<.0001	–

Model 1: unadjusted. Model 2: adjusted for age, gender, and race. Model 3: adjusted for age, gender, race, marital status, education level, poverty-income ratio, smoking status, alcohol use, physical activity (MET/wk), hypertension, diabetes, and hyperlipidemia. Data are presented as odds ratios (OR) with 95% CI. BRI quartiles: Q1 (lowest), Q2, Q3, Q4 (highest). *P* for trend was calculated by treating BRI quartiles as a continuous variable.

BRI = body roundness index, CI = confidence interval, MET = metabolic equivalent of task, MUI = mixed urinary incontinence, OR = odds ratio, SUI = stress urinary incontinence, UUI = urge urinary incontinence.

### 3.3. Nonlinear relationship between BRI and UI

**Figure 2.**
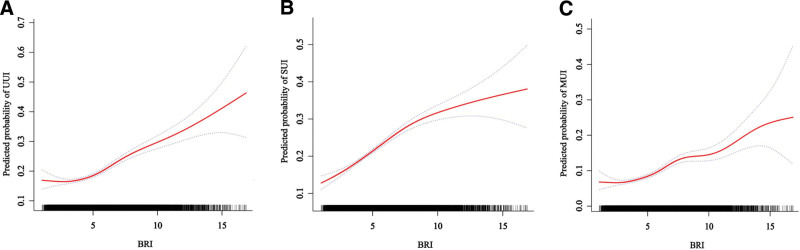
Smooth curve fitting of BRI and risk of UI subtypes. Restricted cubic spline models with 3 knots were used to examine the nonlinear relationship between BRI and the predicted probability of UI subtypes, adjusted for age, gender, race, marital status, education level, poverty-income ratio, smoking status, alcohol use, physical activity, hypertension, diabetes, and hyperlipidemia. The solid red line represents the predicted probability, and the shaded gray area indicates the 95% confidence interval. The distribution of participants across the BRI spectrum is represented by small vertical lines at the bottom of each graph. (A) UUI; (B) SUI; (C) MUI. The x-axis shows BRI values ranging from approximately 1 to 15, while the y-axis shows the predicted probability of each UI subtype. BRI = body roundness index, MUI = mixed urinary incontinence, SUI = stress urinary incontinence, UUI = urge urinary incontinence.

To further clarify the relationship between BRI and UI, we performed smoothed curve fitting analysis (Fig. 2) and threshold effect analysis (Table 3), which revealed distinct nonlinear patterns between BRI and the UI subtypes. For UUI, risk gradually increased with rising BRI values, reaching nearly 0.5 at BRI = 15 (Fig. 2A). Threshold analysis identified an inflection point at BRI = 2.72, with a significant inverse association below this value (OR = 0.71, 95% CI: 0.55–0.91, *P* = .0067) that reversed to a positive association above it (OR = 1.13, 95% CI: 1.11–1.15, *P* < .0001). For SUI, risk increased steadily with BRI until approximately BRI = 10, after which the slope steepened (Fig. 2B). Threshold analysis detected an inflection point at BRI = 7.8, with a stronger association below this threshold (OR = 1.17, 95% CI: 1.14–1.20, *P* < .0001) that became nonsignificant above it (OR = 1.03, 95% CI: 0.99–1.08, *P* = .0979). MUI demonstrated the most pronounced threshold effect, with risk remaining below 0.1 until BRI ≈ 5, followed by an accelerated increase (Fig. 2C). This was confirmed by threshold analysis with an inflection point at BRI = 7.8, showing a stronger association below this value (OR = 1.18, 95% CI: 1.14–1.21, *P* < .0001) that attenuated but remained significant above it (OR = 1.08, 95% CI: 1.03–1.13, *P* = .001). Log-likelihood ratio tests confirmed that the nonlinear threshold models were superior to the standard linear models (all *P* < .05).

**Table 3:** Threshold effect analysis of BRI and UI subtypes.

Variable	SUI		UUI		MUI	
	OR (95% CI)	*P*-value	OR (95% CI)	*P*-value	OR (95% CI)	*P*-value
Model I						
Linear effect of BRI	1.13 (1.11–1.14)	<.0001	1.12 (1.10–1.14)	<.0001	1.14 (1.12–1.16)	<.0001
Model II						
Inflection point (K)	7.8		2.72		7.8	
BRI<K	1.17 (1.14–1.20)	<.0001	0.71 (0.55–0.91)	.0067	1.18 (1.14–1.21)	<.0001
BRI>K	1.03 (0.99–1.08)	.0979	1.13 (1.11–1.15)	<.0001	1.08 (1.03–1.13)	.001
Likelihood ratio test (*P*-value)	<.001	–	<.001	–	.011	–

All models adjusted for: age, sex, race, marital status, education level, poverty-income ratio, smoking status, alcohol consumption, physical activity (MET-min/wk), hypertension, diabetes mellitus, and hyperlipidemia. Data presented as odds ratio (95% confidence interval).

BRI = body roundness index, CI = confidence interval, MET = metabolic equivalent of task, MUI = mixed urinary incontinence, OR = odds ratio, SUI = stress urinary incontinence, UUI = urge urinary incontinence.

### 3.4. Subgroup analysis

**Figure 3.**
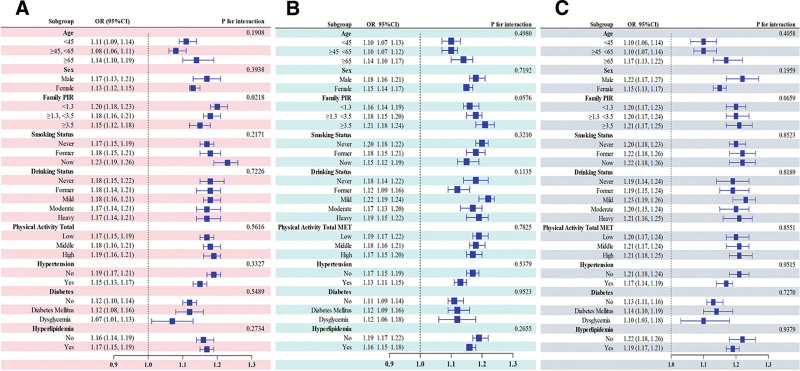
Subgroup investigation regarding the correlation between BRI and 3 categories of UI. (A–C) respectively represent the results of subgroup analyses for the relationships between BRI and SUI, UUI, and MUI in different stratifications. All stratified factors include gender, age, the family poverty-income ratio, smoking status, alcohol use, diabetes, and hypertension, except the stratified factor itself. MET = metabolic equivalent of task, PIR = poverty-income ratio.

Subgroup analyses assessed the robustness of BRI-UI associations (Fig. 3). PIR significantly moderated the association between BRI and SUI, with stronger effects among low-income individuals (*P* = .0218) (Fig. 3A). The PIR moderation for UUI and MUI showed borderline significance (*P* = .0576, *P* = .0659) (Fig. 3B and C). No significant interactions emerged across other demographic and clinical factors (gender, age, smoking, alcohol consumption, physical activity, hypertension, diabetes, and hyperlipidemia; all *P* > .05), demonstrating BRI’s consistent role as a robust risk factor for UI across diverse populations.

## 4. Discussion

In this large-scale population-based cross-sectional study, we investigated the link between BRI and different types of UI in American adults aged 20 and above. Our findings revealed significant positive dose-response relationships between BRI and SUI, UUI, and MUI. In fully adjusted models, each unit increase in BRI was associated with 12%, 12%, and 13% increased risks for the 3 UI types, respectively, with mixed UI showing the strongest association with BRI (OR = 1.13, 95% CI: 1.11–1.16). More importantly, through restricted cubic spline analysis and threshold effect analysis, we discovered nonlinear relationships between BRI and UI, with UUI exhibiting an inflection point at BRI = 2.72, while both SUI and MUI showed threshold effects at BRI = 7.8, indicating that the impact of BRI on each UI subtype may differ at varying levels.

BRI and UI may be affected by specific factors, including the fact that the prevalence of UI tends to rise with age, while the BRI level is also correlated with advancing age.^[24]^ Additionally, hypertension, hyperlipidemia, and diabetes are not only linked to the risk of UI,^[22,23]^ but also closely associated with BRI.^[25,26]^ Therefore, some covariates were included for further subgroup analysis in this study. The results indicated that PIR significantly moderated the association between BRI and SUI (*P* = .0218), with a more pronounced association observed among participants with PIR < 1.3. This may be because the prevalence of abdominal obesity is significantly higher in low-income populations, leading the BRI – an obesity metric centered on abdominal morphology – to more accurately reflect true visceral fat load in this group.^[27,28]^ Consequently, the statistical association between BRI and UI was strengthened in the PIR < 1.3 subgroup, suggesting that socioeconomic factors may play an important role in the development and progression of UI.

Previous studies indicate that obesity, particularly abdominal obesity, is closely associated with the incidence and severity of UI.^[29,30]^ A meta-analysis of 24 prospective studies demonstrated that each 10 cm increase in WC elevates the odds of UI by approximately 14% to 22%, while each 10 kg increase in body weight raises the odds by 11% to 62%.^[31]^ Another large-scale longitudinal study in the Chinese population revealed that overweight or obese patients exhibited lower UI remission rates after 4 years compared to those with normal or low BMI. Furthermore, individuals with BMI ≥ 24 kg/m^2^ who initially presented with pure SUI or UUI had a higher predicted probability of progressing to MUI within 4 years.^[32]^ In our study, patients with any of the 3 types of UI demonstrated higher BRI levels, while those without generally exhibited a tendency toward lower BRI, further reinforcing the link between abdominal obesity and UI. Therefore, intervening in obesity through participation in physical activities, especially improving abdominal obesity, may have a positive effect on the prevention of UI and effectively reduce its risk.

From an anatomical perspective, obesity-induced weight gain may lead to tension in the pelvic floor and bladder muscles. Over time, this additional weight can ultimately cause the pelvic floor muscles to become overstretched and weaken their ability to support urinary continence. Due to weakened pelvic floor muscles, the urethral sphincter may become lax, which increases the likelihood of involuntary urine leakage.^[33,34]^ Furthermore, abdominal obesity may lead to the accumulation of abdominal fat, thereby increasing intra-abdominal pressure. Chronic high intra-abdominal pressure can damage pelvic floor muscles and their innervation, resulting in muscle atrophy and deformation, ultimately causing pelvic floor muscle weakness and dysfunction. The increased intra-abdominal pressure may also exacerbate the laxity of the urethral sphincter, making it more difficult to regulate urine release, which consequently raises the likelihood of UI.^[35,36]^ Pathophysiologically, the accumulation of visceral adipose tissue induced by obesity leads to dysregulation of multiple inflammatory cytokines, such as tumor necrosis factor-α (TNF-α) and interleukin-6 (IL-6), which further activates oxidative stress. This results in metabolic disturbances and impaired collagen metabolism in human pelvic fibroblasts, ultimately increasing the incidence and severity of UI.^[37,38]^ Consequently, accurate assessment of obesity is crucial for screening and implementing early interventions in high-risk populations for UI.

Traditionally, BMI has been the most widely used indicator for assessing obesity and related health risks. Previous studies have shown that higher BMI in adult women is independently associated with increased risk and frequency of UI.^[39,40]^ However, some studies suggest that BMI has certain limitations in differentiating between lean mass and body fat percentage and in evaluating abdominal obesity.^[41–43]^ Abdominal obesity, particularly the accumulation of deep subcutaneous fat, has been identified as a key driver of UI. Consequently, BMI – calculated as weight divided by height squared – may fail to accurately reflect an individual’s obesity status, especially in those with UI. WC is commonly used to assess abdominal obesity, but it cannot capture overall body fat distribution or distinguish between visceral fat and subcutaneous fat.^[44]^ In contrast, the calculation of BRI is based on an elliptical model of body shape, using eccentricity to estimate visceral fat and total body fat percentage, which can more precisely evaluate the body fat distribution and identify abdominal obesity.^[10]^ A meta-analysis revealed that, compared with BMI, body adiposity index, waist-to-hip ratio, and body shape index, BRI was a more effective predictor of metabolic syndrome.^[45]^ In addition, other studies showed that BRI was associated with a variety of diseases, including overactive bladder,^[14]^ sarcopenia,^[46]^ and diabetes.^[26]^ However, the correlation between BRI and UI remains unclear. Our research indicated a significant positive correlation between BRI and UI, suggesting that BRI, a noninvasive and easy-to-manage assessment tool, may serve as an effective indicator for screening high-risk individuals for UI.

This study has several strengths. Firstly, it innovatively explored the association between the novel obesity assessment index, BRI, and UI. Secondly, the study made comprehensive adjustments for demographic characteristics, lifestyle factors, comorbidities, and other potential confounders based on clinical knowledge and previous research, thereby ensuring the credibility of the research results. Thirdly, detailed subgroup analyses further explored the robustness of the relationship between BRI and UI in different populations. Finally, the large sample size obtained from the NHANES database enhanced the reliability and generalizability of the findings.

However, the limitations of this study should not be overlooked. Firstly, the cross-sectional design precludes establishing causal inferences between BRI and UI. Secondly, despite adjustment for multiple covariates, residual confounding from unmeasured factors may still be present. Thirdly, for certain variables, the diagnoses were based solely on self-reported data without objective testing methods to confirm, which may introduce recall bias. Consequently, although our study provided novel evidence supporting the potential of BRI as a screening indicator for the high-risk population of UI, further longitudinal and prospective studies are necessary to validate the findings in the future.

## 5. Conclusion

Our study reveals that BRI is consistently associated with all UI subtypes after adjustment; associations strengthen with higher BRI, most pronounced for MUI, with SUI and UUI increasing earlier; nonlinearity and subtype-specific inflection points are evident; causality is limited by the cross-sectional design; prospective studies are needed to confirm findings and explore mechanisms and subgroup differences.

## Acknowledgments

The authors thank all the participants and researchers of the NHANES databases.

## Author contributions

**Conceptualization:** Haote Chen.

**Data curation:** Tianyu Huang, Qiulu Huang.

**Formal analysis:** Qiulu Huang, Liyang Chang.

**Funding acquisition:** Haote Chen, Yanjuan Li.

**Investigation:** Tianyu Huang.

**Methodology:** Tianyu Huang, Qiulu Huang, Liyang Chang.

**Project administration:** Mei Yang.

**Resources:** Yanjuan Li, Mei Yang.

**Software:** Qiulu Huang.

**Supervision:** Yanjuan Li, Mei Yang.

**Visualization:** Tianyu Huang.

**Writing – original draft:** Tianyu Huang.

**Writing – review & editing:** Qiulu Huang, Haote Chen, Liyang Chang, Yanjuan Li, Mei Yang.
